# Genome-Wide Studies of Rho5-Interacting Proteins That Are Involved in Oxidant-Induced Cell Death in Budding Yeast

**DOI:** 10.1534/g3.118.200887

**Published:** 2019-01-22

**Authors:** Komudi Singh, Mid Eum Lee, Maryam Entezari, Chan-Hun Jung, Yeonsoo Kim, Youngmin Park, Jack D. Fioretti, Won-Ki Huh, Hay-Oak Park, Pil Jung Kang

**Affiliations:** * Department of Molecular Genetics, The Ohio State University, Columbus, OH 43210; † Molecular Cellular Developmental Biology Program, The Ohio State University, Columbus, OH 43210; ‡ Department of Biological Sciences, Seoul National University, Seoul 08826, Republic of Korea

**Keywords:** yeast knockout strains, bimolecular fluorescence complementation, oxidative stress, regulated cell death, autophagy

## Abstract

Rho GTPases play critical roles in cell proliferation and cell death in many species. As in animal cells, cells of the budding yeast *Saccharomyces cerevisiae* undergo regulated cell death under various physiological conditions and upon exposure to external stress. The Rho5 GTPase is necessary for oxidant-induced cell death, and cells expressing a constitutively active GTP-locked Rho5 are hypersensitive to oxidants. Yet how Rho5 regulates yeast cell death has been poorly understood. To identify genes that are involved in the Rho5-mediated cell death program, we performed two complementary genome-wide screens: one screen for oxidant-resistant deletion mutants and another screen for Rho5-associated proteins. Functional enrichment and interaction network analysis revealed enrichment for genes in pathways related to metabolism, transport, and plasma membrane organization. In particular, we find that *ATG21*, which is known to be involved in the CVT (Cytoplasm-to-Vacuole Targeting) pathway and mitophagy, is necessary for cell death induced by oxidants. Cells lacking Atg21 exhibit little cell death upon exposure to oxidants even when the GTP-locked Rho5 is expressed. Moreover, Atg21 interacts with Rho5 preferentially in its GTP-bound state, suggesting that Atg21 is a downstream target of Rho5 in oxidant-induced cell death. Given the high degree of conservation of Rho GTPases and autophagy from yeast to human, this study may provide insight into regulated cell death in eukaryotes in general.

Rho GTPases regulate diverse cellular processes in species ranging from yeast to humans. In addition to their functions in cytoskeleton organization in various cell types (Hall 2012), Rho GTPases including Rac have been implicated in phagocyte killing and programmed cell death (PCD) in *Caenorhabditis elegans* (Ravichandran and Lorenz 2007). The evolutionally conserved role of Rac during the engulfment of apoptotic cells in mammals and *C. elegans* is related to their critical roles in cytoskeleton reorganization (Ravichandran and Lorenz 2007). Rac GTPases are also involved in activation of NADPH oxidases (NOX enzymes), which accept electrons from NADPH to produce the superoxide radical, in neutrophils and non-phagocytic cells (Abo *et al.* 1991; Pick 2014; Werner 2004). Budding yeast has nine ORFs with sequence similarity to mammalian NADPH oxidases, and some of them are involved in the regulation of the actin cytoskeleton (Rinnerthaler *et al.* 2012), but their link to Rho GTPase is not known.

A large number of studies have shown that yeast cells undergo ‘regulated cell death (RCD)’ or PCD under various physiological conditions (Carmona-Gutierrez *et al.* 2018; Strich 2015). Regulation of cell death appears conserved in yeast, sharing some common regulators of cell death in metazoan and other multicellular systems, including the AAA-ATPase Cdc48/VCP (Madeo *et al.* 1997; Braun and Zischka 2008; Braun *et al.* 2006) and metacaspases (Madeo *et al.* 2002). Yet the mechanisms by which yeast cell death is regulated are not well understood. We previously found that Rho5, which is closely related to Rac GTPases in mammals, is necessary for oxidant-induced cell death in budding yeast (Singh *et al.* 2008). Since Rho5 interacts with Trr1, thioredoxin reductase, specifically in its active GTP-bound state, we proposed that Rho5 might downregulate the thioredoxin anti-oxidant system during cell death (Singh *et al.* 2008). Other studies have suggested that Rho5 downregulates the yeast cell wall integrity pathway (Schmitz *et al.* 2002) and is involved in osmotic stress response (Annan *et al.* 2008), although the underlying mechanisms are not clear. Consistent with these previous reports, cells lacking *DCK1* and *LMO1*, the homologs of mammalian DOCK180 and ELMO, which catalyze the nucleotide exchange of Rac1 or Cdc42 (Brugnera *et al.* 2002; Cote and Vuori 2002), exhibit hyper-resistance to cell wall stress and hydrogen peroxide (H_2_O_2_) (Schmitz *et al.* 2015).

Since Rac is an important player during apoptotic cell death in other cell types, we asked whether a similar mechanism might be involved in Rho5-mediated cell death in yeast. The *rho5^G12V^* mutant, which is believed to encode the GTP-locked Rho5 *in vivo*, exhibits higher sensitivity to oxidants compared to cells lacking *TRR1* (Singh *et al.* 2008), suggesting that Rho5 has additional targets to promote cell death. We thus performed genome-wide screens to identify genes that are closely associated with Rho5 and are likely involved in oxidant-induced cell death. Here, we report that several genes involved in vesicular traffic and organelle organization are important for oxidant-induced cell death. In particular, we found that *ATG21*, which is known to be involved in the CVT pathway and mitophagy (Strømhaug *et al.* 2004), is necessary for cell death mediated by Rho5.

## Materials And Methods

### Plasmids, yeast strains, and growth conditions

The *Saccharomyces cerevisiae* haploid knockout (YKO) strains (Thermo Scientific Open Biosystem) and wild-type (WT) BY4741 were used to screen for deletion mutants that were resistant to oxidants. A collection of VN (the N-terminal fragment of Venus, a yellow fluorescent protein)-tagged yeast strains (Sung *et al.* 2013) was used to screen for Rho5-binding proteins by bimolecular fluorescence complementation (BiFC) assays. All yeast strains and plasmids used in this study are listed in Supplemental Table S1 and S2, respectively, with a brief description. Standard methods of yeast genetics, DNA manipulation, and growth conditions were used (Guthrie and Fink 1991). Yeast strains were grown in rich yeast medium YPD (yeast extract, peptone, dextrose) or synthetic complete (SC) containing 2% dextrose as a carbon source, unless stated otherwise.

### Growth phenotype and treatment With H_2_O_2_ or heat stress

Sensitivity to H_2_O_2_ was monitored by plating assays, as previously described (Singh *et al.* 2008). Since the laboratory WT strains exhibited a varying degree of sensitivity to H_2_O_2_ depending on the background (Singh *et al.* 2008), 1 mM H_2_O_2_ was used for all strains in BY4741 background, whereas 3∼4 mM H_2_O_2_ was used for strains in HPY210 background to test oxidant-induced cell death. All strains were examined in comparison to WT in the isogenic strain background. Treatment with ramped heat stimulus was performed as previously described (Teng *et al.* 2011): cells in fresh culture (OD_600_ = 0.5∼0.6) were treated by increasing temperature in a PCR machine, first from 25° to 40° over 2 min and then 40° to 51° during the 10 min period. Cells were then kept at 51° for 10 min and plated on YPD. Cells grown for 1 day (postdiauxic) were treated by increasing temperature from 25° to 55° during the 15 min period and then kept at 55° for 10 min before plating on YPD.

### Cell viability assays

Plasma membrane integrity was monitored by staining cells with propidium iodide (PI) immediately after incubation with 4 mM H_2_O_2_ for 4 hr (or mock-treated), as previously described (Kainz *et al.* 2017). Single z-stack images of PI-stained cells were captured using a Nikon E800 microscope with a 40x objective lens and TRITC/TexasRed filter from Chroma Technology, Hamamatsu ORCA-2 CCD (Hamamatsu Photonics), and Slidebook software (Intelligent Imaging Innovations). The methylene blue reduction test was used to determine metabolically active cells, which convert methylene blue to colorless leucomethylene blue (Bapat *et al.* 2006). After H_2_O_2_ treatment (or mock-treated) as described above, cells were stained with 10 μM methylene blue (Sigma) and then observed by DIC (Differential interference contrast microscopy) using the Nikon E800 microscope with a 100x/1.30 NA oil-immersion objective lens. The same H_2_O_2_-treated and mock-treated cells were also plated on YPD plates (∼200 cells per plate) to determine the colony forming unit (CFU).

### Genome-wide screen for H_2_O_2_-resistant mutants

First, the YKO collection in 96-well plates was transformed with a multi-copy rho5^G12V^ plasmid to generate a ‘sensitized’ strain collection, and the transformants were selected on SC-Ura plates. Each transformant as well as WT strain carrying the same plasmid organized in the 96-well plates (in duplicate sets) were treated with 1 mM H_2_O_2_ for 3 hr or mock-treated, and then plated on SC-Ura plates using a 96-pin metal multi-blot replicator (‘Frogger’, V&P Scientific, Inc). After two days of incubation at 30°, deletion mutants that were more resistant to H_2_O_2_ than WT were visibly identified. Those potential candidate strains were subjected to retesting under the same conditions except by growing them in individual liquid culture using SC-Ura medium. The authenticity of each deletion that was reproducibly resistant to H_2_O_2_ were then tested by genomic PCR. Second, deletions of these candidate genes were generated in another strain background (HPY210) and tested for resistance to oxidants with 4 mM H_2_O_2_ (see above).

### Genome-wide screen by BiFC assays

Briefly, a haploid strain HY1029, which expresses VC (the C-terminal fragment of Venus) fused to the N terminus of Rho5, was mated with each VN fusion strain in 96-well plates, followed by selection of diploids on SC-Met-Lys, as previously described (Sung *et al.* 2013)(see Table S1). Diploid cells were grown to mid-logarithmic phase at 30° in SC medium, and initial screens were performed using 96-well glass-bottomed microplates (MGP096, Matrical Bioscience) and a Nikon Eclipse E1 microscope and a Plan Fluor 100×/1.30 NA oil immersion objective. Fluorescence images were obtained by a PhotoFluor LM-75 light source (89 North Inc.).

### Microscopy and image analysis

Since the same split site (154/155) was used to generate both EYFP and Venus truncated forms for BiFC, candidate VN fusions from the initial screen were tested for nucleotide-specific interactions with Rho5 using YFP^C^ (the C-terminal fragment of YFP) fusions of WT and Rho5 mutant proteins. Cells were grown in the appropriate synthetic medium overnight and then freshly sub-cultured for 3∼4 hr in the same medium prior to imaging. For images shown in Figure S2, 3 z-stack (0.4 μm step) images were captured at room temperature (23∼25°) using a Nikon E800 microscope fitted with a 100x/1.30 NA oil-immersion objective lens and a YFP filter from Chroma Technology (see above). Out-of-focus signals were removed by deconvolution using Slidebook software (Intelligent Imaging Innovations). For images shown in Figure 5A, slides were prepared similarly on an agarose slab, and 3 z-stack (0.4 μm step) images were captured at 22° using a spinning disk confocal microscope (Ultra-VIEW VoX CSU-X1 system; Perkin Elmer-Cetus) equipped with a 100x /1.4 NA Plan Apochromat objective lens (Nikon); 440-, 488-, 515- and 561-nm solid-state lasers (Modular Laser System 2.0; Perkin Elmer-Cetus); and a back-thinned EM CCD (ImagEM C9100-13; Hamamatsu Photonics) on an inverted microscope (Ti-E; Nikon).

Image processing and analyses were performed using ImageJ (National Institutes of Health). Summed intensity projections of z stacks of representative images were used to generate Figure 5Aa. To quantify BiFC signals, summed intensity projections were analyzed after background subtraction as follows: First, a fluorescence threshold was set above background that was estimated from a control strain (that expressed untagged Rho5 together with Atg21-VN). This threshold mainly selected fluorescent pixels on the vacuolar membrane. About 40∼62% of cells expressing YFP^C^-Rho5 or YFP^C^-Rho5^G12V^ had YFP signals above threshold (n = 86 for WT; n = 156 for Rho5^G12V^). In contrast, less than 10% of cells expressing YFP^C^-Rho5^K16N^ (n = 142) had YFP signal above threshold. To quantify BiFC signals in individual cells, all selected pixels above threshold in each cell were measured by drawing ROI around the cell boundary (based on the synchronized DIC image). The same threshold was also applied to all three strains that were captured and processed using the same conditions. Average integrated density of all ROIs (depicted with each mark on the graph) was calculated for each image set, and mean ± SEM from analyses of 4 independent image sets are shown in Figure 5Ab.

### Yeast two-hybrid assay

A two-hybrid assay was carried out as previously described (Gyuris *et al.* 1993). Atg21 was expressed as an activation domain (AD) fusion protein using plasmid pJG4-5-ATG21 (see Table S2). WT and mutant Rho5 proteins were expressed as DNA-binding domain (DBD) fusions using plasmids pEG202-RHO5, pEG202-rho5^G12V^, and pEG202-rho5^K16N^, all of which also carry the C328S substitution, as previously described (Singh *et al.* 2008). The yeast strain EGY48 carrying the *LEU2* reporter gene was transformed with pJG4-5-ATG21 and each of pEG202-RHO5 plasmids (or empty vector controls), and several independent transformants from each transformation were plated on SC-Gal plates lacking Leu (and Trp and His to maintain the plasmids) to test the Rho5-Atg21 interaction. To compare the levels of WT or mutant Rho5 proteins fused to LexA, yeast extracts were prepared from the strain (EGY48) carrying each pEG plasmid and then were subjected to immunoblotting with rabbit polyclonal anti-LexA antibodies (EMD Millipore) and Alexa Fluor 680 goat anti-rabbit IgG (LI-COR Biosciences).

### Gene Ontology (GO) term enrichment analysis

Pathway enrichment analyses of the lists of genes derived from genome-wide studies were performed using the DAVID (Database for Annotation, Visualization and Integrated Discovery) bioinformatics resources (Huang da *et al.* 2009a, 2009b). The GO for biological processes (BP), molecular functions (MF), cellular components (CC), and Kyoto Encyclopedia of Genes and Genomes (KEGG) pathways that were significantly enriched are shown with respective p values (corrected for multiple comparison by Benjamini) in Figure 3C and Tables S3–S5.

### Statistical analysis

Data analyses and graph plotting were performed using Prism 6 (GraphPad Software). Mean and SEM (error bars) are provided in the bar graphs. A two-tailed student’s *t*-test was performed to determine statistical differences between two sets of data: ns (not significant) for *P* ≥ 0.05; *P < 0.05; **P < 0.01; ***P < 0.001.

### Data availability

Strains and plasmids are available upon request. The VN fusion library is distributed by the Bioneer Corp (www.bioneer.com). The authors affirm that all data necessary for confirming the conclusions of the article are present within the article, figures, and the supplemental files. Tables S1 and S2 contain lists of yeast strains and plasmids used in this study. Tables S3–S5 include all genomics data and GO term enrichment analyses by DAVID. Figures S1 shows spot assays of selected deletion mutants to test resistance to H_2_O_2_. Figure S2 shows BiFC analyses of candidate VN fusions with YFP^C^ fusions of WT or mutant Rho5. Supplemental material available at Figshare: https://doi.org/10.25387/g3.7294976.

## Results And Discussion

### Rho5 is necessary for cell death upon exposure to oxidants and heat stress

**Figure 1:**
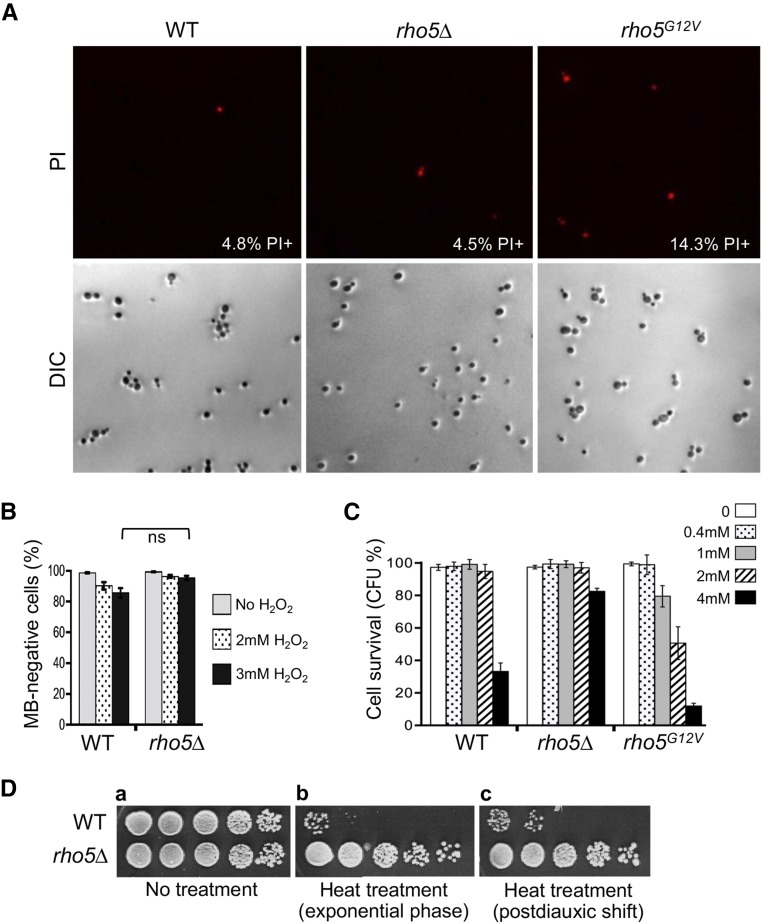
Rho5 mediates regulated cell death triggered by H_2_O_2_ or heat ramp. A. PI staining of each strain (HPY210 background) right after exposure to 3 mM H_2_O_2_ for 4 hr. Average percentage of PI^+^ cells are indicated from three sets of data (n = 100∼200 for each sample per test). Mock-treated cells showed low numbers of PI^+^ cells (2∼3.5%) for all strains (not shown). B. MB staining of each strain (HPY210 background) after exposure to H_2_O_2_ treatment for 4 hr or mock-treated. Mean ± SEM (error bars) are shown from three sets of data (n = 100 for each sample per test). C. CFU was measured for each strain (HPY210 background) after H_2_O_2_ treatment for 4 hr. Mean ± SEM (error bars) are shown from three sets of data (n = 100 for each sample per test) D. Cell survival was determined by a fivefold serial dilution of each strain (BY4741 background) grown in YPD after subjecting to heat ramp: (a) cells without treatment; (b) cells from fresh cultures (OD_600_ = 0.5∼0.6) (b); and (c) cells grown 1d (postdiauxic).

While oxidant-induced cell death depends on Rho5 in budding yeast (Singh *et al.* 2008), it remained unclear how Rho5 promotes cell death. Since yeast medium generally contains high levels of antioxidants, typically a higher concentration of H_2_O_2_ is used to introduce oxidative stress in budding yeast than other cell types. In addition, the sensitivity to H_2_O_2_ differs significantly depending on strain background (Singh *et al.* 2008). To investigate regulated cell death and to avoid accidental cell death caused under harsh conditions, it was thus critical to establish a proper experimental condition for each strain background. To ensure the Rho5-mediated cell death conditions, we characterized *rho5* mutants by staining with PI or methylene blue (MB) immediately after treating with H_2_O_2_ and also determined CFU following the same treatment with H_2_O_2_. PI is highly charged and therefore normally cell impermeant, but it penetrates damaged membranes (Stan-Lotter *et al.* 2006). Prior to H_2_O_2_ treatment, less than 3% of WT, *rho5*Δ, and *rho5^G12V^* cells were PI-positive (data not shown), and the percentage of PI-positive cells was slightly increased in the *rho5^G12V^* cells after treatment with H_2_O_2_ (Figure 1A). Similarly, there was only a minor difference in the percentage of MB-negative cells between WT and *rho5*∆ (Figure 1B), suggesting the majority of cells have metabolic activity immediately following the treatment with H_2_O_2_. Yet the CFU was significantly different among these strains: while over 80% of *rho5*Δ cells were viable, less than 10% of *rho5^G12V^* cells and 30% of WT cells were viable upon treatment with 4 mM H_2_O_2_ (Figure 1C). Thus, the majority of these cells likely entered early apoptotic stage (PI^-^) immediately upon exposure to H_2_O_2_, and cell death likely occurred during subsequent incubation without further stress. We observed that a lower H_2_O_2_ concentration (that caused cell death to a majority of WT cells in S288C background) had little effect on cell survival in the HPY210 background (compare Figures 1C & 5C).

A previous report has shown that a ramped heat stimulus, rather than sudden heat shock, results in reproducible gene-specific survival phenotypes (Teng *et al.* 2011). By following a similar procedure, we tested viability of *rho5*Δ when subjected to a controlled heat ramp (see Materials and Methods). We found that the *rho5*Δ mutant was more resistant to heat stress than WT during the exponential phase of growth and postdiauxic shift (Figure 1D). These observations suggest that Rho5 promotes apoptosis upon exposure to various stresses in addition to oxidants, consistent with previous studies (Schmitz *et al.* 2002; Singh *et al.* 2008; Annan *et al.* 2008; Schmitz *et al.* 2015).

### Cell death mediated by Rho5 may be independent of known apoptotic factors in budding yeast

**Figure 2:**
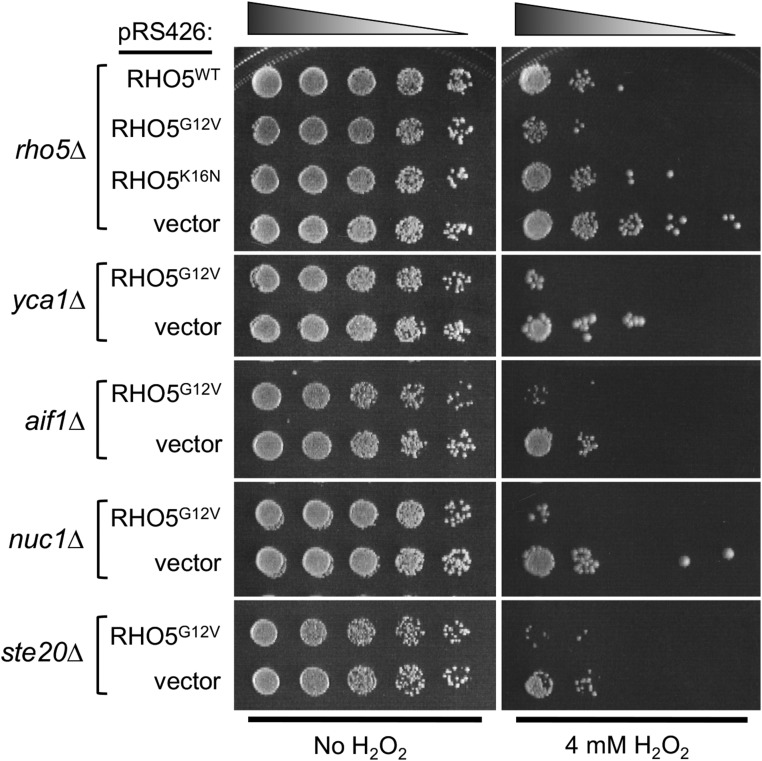
The GTP-locked Rho5 promotes cell death independently of known apoptotic factors in budding yeast. Each indicated deletion strain (in HPY210 background) carrying a multicopy RHO5 plasmid or vector control, as indicated, was treated with 4 mM H_2_O_2_ for 4 hr (or mock treated), and then fivefold serial dilutions (starting from OD_600_ = 1) were plated on SC-Ura plates.

A number of orthologs of mammalian genes involved in apoptosis have been implicated in RCD in budding yeast. These include *YCA1* (metacaspase) (Madeo *et al.* 2002), *AIF1* (an ortholog of mammalian Apoptosis-Inducing Factor) (Wissing *et al.* 2004), *NUC1* (mitochondrial nuclease, an ortholog of mammalian endoG) (Büttner *et al.* 2007), and *STE20*, a PAK (p21-activated protein kinase) (Ahn *et al.* 2005). We asked whether cell death mediated by Rho5 involves these genes. If any of these gene products functions downstream of Rho5 in the same oxidant-induced cell death pathway, we would expect that cells lacking such a gene might be resistant to oxidants even when Rho5^G12V^ was expressed, which caused hypersensitivity to oxidants (Singh *et al.* 2008). We thus tested deletion mutants of these apoptotic factors after transforming with a plasmid carrying *rho5^G12V^* or a vector control. Plate assays using serial dilutions of these transformants indicated that the expression of the GTP-locked Rho5 caused cells to be severely sensitive to H_2_O_2_ in these mutants (Figure 2). In addition, all these deletion mutants (which were in the same genetic background as *rho5*Δ) with the vector control were not as resistant to H_2_O_2_ as *rho5*Δ. These observations suggest that Rho5 promotes cell death independently from these previously known players in yeast cell death.

### Genome-wide screen uncovers potential downstream mediators of Rho5 involved in oxidant-induced cell death

**Figure 3:**
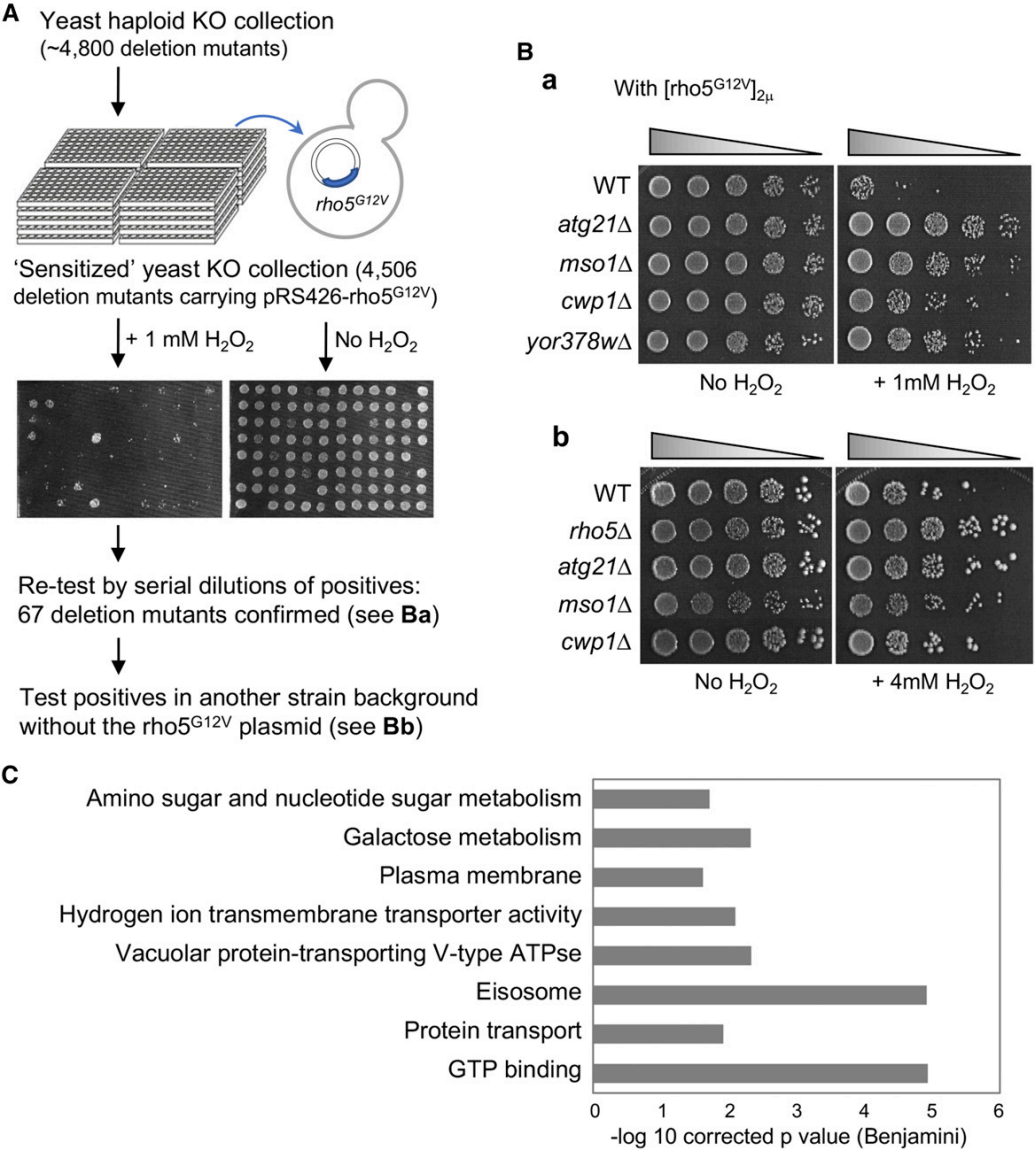
A genome-wide screen for deletion mutants that are resistant to H_2_O_2_. A. A scheme of the screen for deletion mutants that are resistant to H_2_O_2_ in a Rho5-dependent manner. A ‘sensitized’ YKO collection was created by transforming with pRS426-rho5^G12V^ and then subjected to H_2_O_2_ treatment or mock-treatment. B. Fivefold serial dilutions of representative deletion mutants resistant to H_2_O_2_: (a) deletion mutants (BY4741 background) carrying pRS426-rho5^G12V^, and (b) deletion mutants (HPY210 background) without the plasmid. C. Enriched GO terms from the ‘sensitized’ YKO screen.

To identify additional players that function together with Rho5 in oxidant-induced cell death, we carried out a genome-wide screen for mutants that failed to undergo cell death upon exposure to H_2_O_2_. To facilitate identification of genes that function downstream of Rho5 (rather than those that function in oxidant-induced cell death independently of Rho5), we generated a collection of ‘sensitized’ deletion mutants by transforming the *rho5^G12V^* plasmid into each deletion strain in the haploid YKO collection. A total of 4,506 deletion strains in the ordered arrays that carried the *rho5^G12V^* plasmid were successfully recovered and then screened for their resistance to H_2_O_2_ (Figure 3A).

From the primary screen of the ‘sensitized’ strain collection, we initially identified 235 candidates that were more resistant to H_2_O_2_ than the WT control strain carrying the same plasmid. We tested serial dilutions of each primary candidate without the *rho5^G12V^* plasmid and then confirmed correct ORF deletions of those that were reproducibly resistant to H_2_O_2_. We also tested the phenotype of additional candidates from those identified from a screen by BiFC assays (see below). Collectively, we identified 67 mutants that were more resistant to H_2_O_2_ than WT. To confirm that the phenotype resulted from the expected deletion (and not any other mutation in the strain background), we generated a deletion of each candidate gene in another strain background and then tested its resistance to H_2_O_2_. Many deletion mutants in this second test exhibited only minor or little resistance to H_2_O_2_ (see Figure 3Bb). The commonly used strain S288C (in which YKO was generated) has a mutated copy of *HAP1* (Gaisne *et al.* 1999), which is involved in carbon catabolite activation of transcription and also gene expression in response to the levels of heme and oxygen (Guarente *et al.* 1984). S288C also has a variant allele of *MIP1*, which increases petite frequency (Dimitrov *et al.* 2009). These factors might have contributed to hypersensitivity of the YKO strains to oxidants. Together from this secondary test, we identified 31 genes whose deletion confers resistance to H_2_O_2_ (Figure S1; Table S3).

To gain insight into the biological processes that were represented in the genes identified from our screen, we then performed pathway enrichment analysis using the DAVID bioinformatics resources (Huang da *et al.* 2009a) (see Materials and Methods). GO terms and KEGG pathways that were significantly enriched could be grouped into main categories of processes associated with GTP binding, intracellular trafficking and the plasma membrane, and metabolism (Figure 3C; Table S3). These results suggest that Rho5 may integrate signals from various intracellular subcompartments to mediate oxidant-induced cell death.

### Genome-wide screen by BiFC assays uncovers potential targets of Rho5

**Figure 4:**
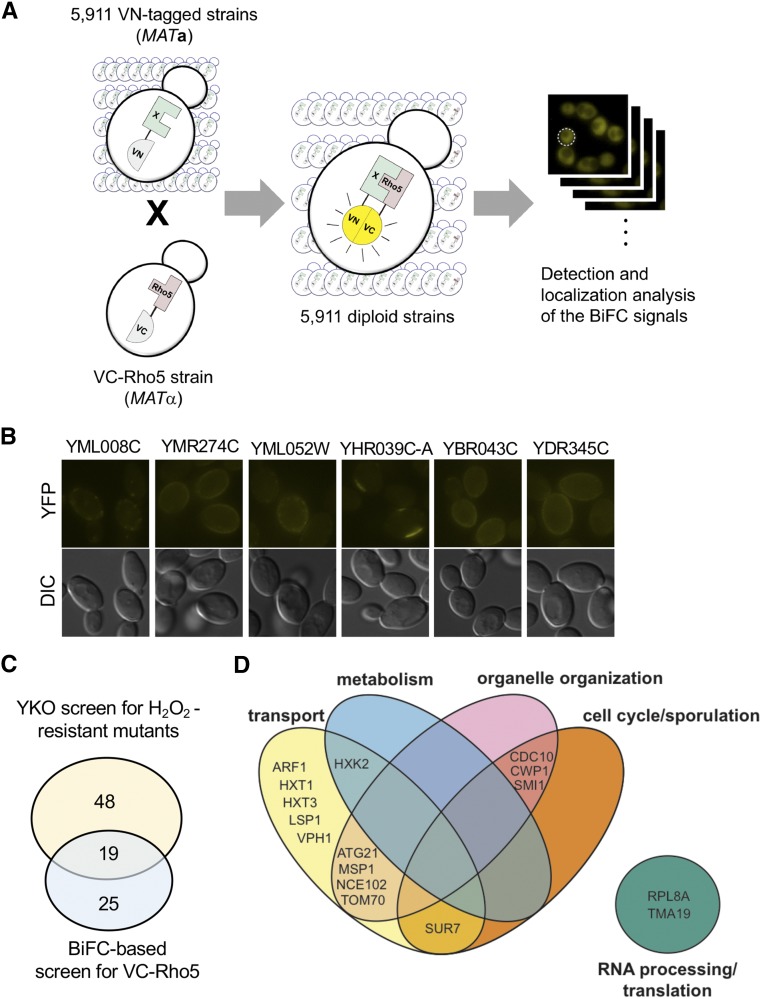
A genome-wide screen for Rho5 interactome by BiFC assays. A. A scheme of the screen of VN fusion library by BiFC assays. B. Examples of positive candidates identified by the BiFC-based screen with VC-Rho5. C. Venn diagram depicting the number of candidate genes identified from two genome-wide screens. D. Venn diagram depicting functional groups enriched from both genome-wide screens.

In parallel to the screen of the yeast deletion library described above, we carried out another genome-wide screen by BiFC assays to identify proteins associated with Rho5. We screened the diploid library in ordered arrays, which were generated by mating each VN strain in the VN fusion library (Sung *et al.* 2013) with a strain expressing VC-Rho5 (Figure 4A). In addition, we tested those positives that were identified only from the screen of the deletion library (see above) by BiFC assays. Collectively, we identified 44 genes, whose VN fusions exhibited positive BiFC signals (Figure 4B; Table S4), including *ATG21* and *CWP1*, which were also identified from the screen for H_2_O_2_-resistant mutants. These positive candidates may associate closely with Rho5, although they may be necessarily involved in oxidant-induced cell death.

To uncover the cellular processes that Rho5 may control via these potential target genes, we performed pathway enrichment analysis and found that the 44 genes identified from the BiFC-based screen were significantly enriched in pathways associated with metabolism, cell wall processes, and intracellular trafficking (Table S4). When we compared genes identified from both genome-wide screens, we found 19 genes that were positive in both categories (Figure 4C), with gene enrichment (with *P* < 0.05) in transport and organelle organization (Figure 4D; Table S5). Our initial screen with VN-Rho5, instead of VN-Rho5^G12V^, might have missed a specific Rho5 effector that might interact transiently with the GTP-bound Rho5. However, BiFC assays facilitate detection of transient protein-protein interactions often by resulting in irreversible bimolecular fluorescent complex formation (Miller *et al.* 2015), and thus a Rho5 effector could be easily trapped with Rho5-GTP. Some of these candidates exhibited GTP-dependent interactions with Rho5, while others appeared less specific to the GTP-bound state of Rho5 (see below).

### A subset of positives identified from both genome-wide screens may interact with Rho5 preferentially in its GTP-bound state

**Figure 5:**
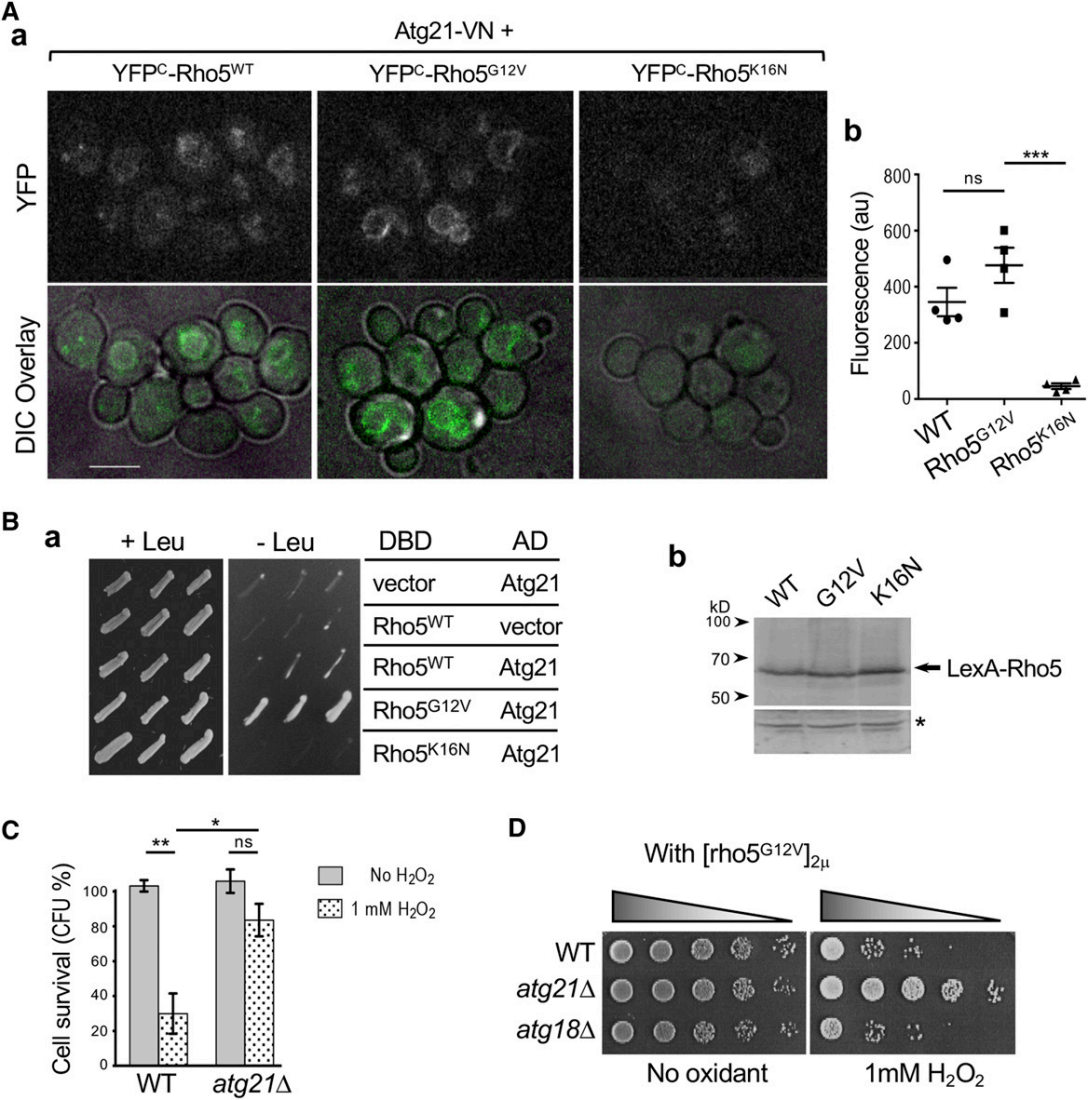
Rho5 interacts with Atg21 preferentially in its GTP-bound state. A. BiFC analyses of strains expressing Atg21-VN together with YFP^C^ fusion of WT or mutant Rho5. (a) Representative images (summed intensity projections) are shown for each strain as indicated. Size bar = 5 μm. (b) Quantification of YFP signals (mean ± SEM) from each BiFC analysis is plotted from 4 independent image sets. Each mark represents average fluorescent intensity from individual cells above threshold (n = 10 ∼25 for each mark in case of WT and Rho5^G12V^; n = 2∼3 for each mark in case of Rho5^K16N^) (see Materials and Methods). Student’s *t*-tests were used and depicted with the following notation: ns (not significant) for *P* ≥ 0.05 and ***P < 0.001. B. (a) Yeast two-hybrid analyses of strains expressing a DBD fusion of WT or mutant Rho5 (or vector control) together with AD-Atg21 or vector control, as depicted. Three independent transformants of each plasmid combination were patched on SGal-Leu (also lacking Trp and His) to monitor interactions between Rho5 and Atg21 (right) and on SGal+Leu (lacking Trp and His) as a control (left). (b) Immunoblot showing LexA fusions to the WT and mutant Rho5 proteins (marked with an arrow) detected with polyclonal antibodies against LexA; an asterisk marks a non-specifically cross-reacting protein as a loading control. C. Cell survival of WT and *atg21*Δ cells (in BY4741 background) after treating with 1 mM H_2_O_2_ for 4 hr or mock treatment. Student’s *t*-tests were used and depicted with the following notation: ns (not significant) for *P* ≥ 0.05; *P < 0.05; and **P < 0.01. D. Fivefold serial dilutions of WT, *atg21*Δ, and *atg18*Δ (all in BY4741 background) carrying pRS-rho5^G12V^ plasmid were spotted after treatment with 1 mM H_2_O_2_ for 4 hr or mock treatment.

Since small GTPases often interact with their downstream targets in a GTP-dependent manner, we tested by BiFC analyses whether the 16 candidates that were identified from both genome-wide screens associate with Rho5 in a nucleotide-specific manner. By examining strains expressing each candidate VN fusion together with YFP^C^-fusions of WT or mutant Rho5, we identified seven VN fusions that exhibited stronger fluorescence signals with YFP^C^-Rho5^G12V^or YFP^C^-Rho5^WT^ compared to YFP^C^-Rho5^K16N^, which is in either GDP-locked or nucleotide-empty state *in vivo* (see Figures 5 & S2), as discussed below. Since YFP^C^ fusions of WT and mutant Rho5 proteins are expressed at about the same level (Singh *et al.* 2008), these observations suggest that these 7 candidates interact preferentially with Rho5-GTP.

Interestingly, a major group of genes that were identified from both genome-wide screens included eisosome components. Eisosomes are protein-based structures associated with specific lipid domains on the plasma membrane furrows, which are known as the MCC (membrane compartment containing Can1) domains in yeast (Walther *et al.* 2006; Karotki *et al.* 2011; Douglas and Konopka 2014). Lsp1, a core component of eisosome (Walther *et al.* 2006), showed positive BiFC signals with WT Rho5 and Rho5^G12V^ but little signal with Rho5^K16N^. Other proteins closely associated with eisosomes, Sur7 and Nce102, also exhibited BiFC signals with Rho5, although their interactions were not specific to its nucleotide-bound state (Figure S2). We found that deletion mutants of the eisosome components, *lsp1*Δ, *nce102*Δ, *sur7*Δ, and *pil1*Δ, were resistant to cell death, albeit moderately, when exposed to oxidants or when subjected to controlled heat ramp (Figure S1, A & B). These findings suggest that Rho5-GTP might signal to eisosomes via Lsp1 under the conditions triggering apoptotic cell death.

Potential involvement of eisosome components in Rho5-mediated cell death is surprising, because previous studies in yeast and other fungi have suggested that the components of the MCC/eisosomes may play multiple roles in resistance to stress (Foderaro *et al.* 2017). Another previous study, however, also reported that deletion of *PIL1* and *LSP1* enhances heat stress resistance (Zhang *et al.* 2004), consistent with our findings. Although their molecular functions are controversial, recent studies have revealed that eisosome proteins play a role in the control of signaling pathways, especially TORC2 activation (Fröhlich *et al.* 2014; Kabeche *et al.* 2014; Berchtold *et al.* 2012). TORC2 serves as a stress sensor to induce various cellular responses including actin polarization, sphingolipid biosynthesis, and stress-related gene transcription (Loewith and Hall 2011). Given the possibility of eisosomes as a link between different signaling pathways, it will be interesting to further investigate the involvement of eisosome proteins in Rho5 signaling pathway.

Cwp1 is another potential player in Rho5-mediated cell death pathway. Interestingly, *CWP1* was previously identified as one of the genes that were essential for cell death induced by the expression of human apoptosis initiator caspase-10 in budding yeast (Lisa-Santamaría *et al.* 2012). The Cwp1-Rho5 BiFC signals appeared at endomembranes including the endoplasmic reticulum (ER) (Figure S2), consistent with the localization of Cwp1-GFP and GFP-Rho5 (Tkach *et al.* 2012; Huh *et al.* 2003; Singh *et al.* 2008) but not at the cell wall or birth scar as reported (Smits *et al.* 2006). However, the location of BiFC signals may not necessarily reflect the site of interaction of the two proteins because of the possibility of irreversible bimolecular fluorescent complex formation. Further studies are required to validate the subcellular location where these proteins interact with each other and to determine the functional significance of this interaction.

Other candidates that exhibit GTP-dependent BiFC signals with Rho5 include Msp1 and Tom70 (Figure S2), which act in sorting and transport of mitochondrial proteins (Nakai *et al.* 1993). Since the mitochondrion is an intracellular platform integrating cell death and energy production (Eisenberg *et al.* 2007; Balaban *et al.* 2005), it will be interesting to investigate further to understand the physiological significance of their interactions with Rho5. Vph1 and Smi1 (also known as Knr4) displayed GTP-dependent BiFC signals with Rho5 (Figure S2), although their deletions exhibited relatively mild resistance to H_2_O_2_ (Figure S1). Vph1 is a subunit of vacuolar ATPase V_0_ domain, which is located on the vacuolar membrane (Manolson *et al.* 1992). Since V-ATPase has been shown to be involved in activation of TORC1 in response to cytoplasmic pH, which in turn depends on glucose (Dechant *et al.* 2014), this result suggests a possible link between Rho5 and TORC1 signaling. *SMI1* encodes a protein localized to the bud neck with a proposed role in coordinating cell cycle progression with cell wall integrity (Martin-Yken *et al.* 2003), but any functional link between Smi1 and Rho5 remains unknown.

Unlike these candidates discussed above and Atg21 (see below), several other candidates did not exhibit a preferential interaction with Rho5-GTP (see Figure S2). While these proteins may possibly interact with a Rho5 domain that does not undergo a drastic conformational change depending on its GDP- or GTP-bound state, further investigation is necessary to validate these interactions with Rho5 *in vivo*. Nevertheless, it is interesting to find that genes involved in glucose metabolism are enriched in our screens. Hxt1 and Hxt3 are low affinity glucose transporters (Lewis and Bisson 1991; Ozcan and Johnston 1999), and Hxk2, which is a predominant hexokinase during yeast growth on glucose, is involved in regulation of expression of *HXT1* (Ozcan and Johnston 1999; Johnston 1999). A large number of studies have shown that glucose metabolism can regulate cell death in variety of cell types (Gottlob *et al.* 2001; Zhao *et al.* 2008a; Zhao *et al.* 2008b; Rathmell *et al.* 2003; Matsuura *et al.* 2016). Interestingly, a recent study suggests that Rho5 relocates from the plasma membrane to mitochondria upon glucose starvation (Schmitz *et al.* 2018). Although the functional significance of this observation is yet to be determined, it is tempting to speculate that there may be a crosstalk between Rho5-mediated cell death pathway and glucose signaling.

### Rho5 may promote cell death via Atg21 Under oxidative stress

Among the candidates that might function in Rho5-mediated cell death, we looked at Atg21 more closely because *atg21*Δ exhibited strong resistance to H_2_O_2_ to a similar extent as *rho5*Δ in two strain backgrounds used in this study and even when Rho5^G12V^ was expressed (see Figures 3Bb, 5C & 5D). BiFC assays with specific Rho5 fusions suggested that Atg21 interacts preferentially with Rho5-GTP. The BiFC signal was mainly observed on the vacuolar membrane with some puncta near and/or on the vacuolar membrane (Figure 5Aa), consistent with the localization of Atg21 (Strømhaug *et al.* 2004) and a part of Rho5 localization (Singh *et al.* 2008). Quantification of the BiFC signals indicated that there were strong fluorescence signals in cells expressing YFP^C^-Rho5^G12V^ and slightly weaker signals in cells expressing YFP^C^-Rho5^WT^, whereas only minor background level of fluorescence was observed in cells expressing YFP^C^-Rho5^K16N^ (Figure 5Ab).

As an alternative way to test the GTP-dependent interaction between Rho5 and Atg21, we performed a yeast two-hybrid assay. An AD-Atg21 fusion was expressed together with a DBD fusion of WT or mutant Rho5 in a strain carrying the *LEU2* reporter. Growth of the cells expressing AD-Atg21 specifically with DBD-Rho5^G12V^, but not with DBD-Rho5^K16N^, on a plate lacking Leu indicated that Rho5-GTP interacts with Atg21 (Figure 5Ba), despite the similar level of DBD fusions of WT and mutant Rho5 proteins (Figure 5Bb). The positive interaction between Rho5^WT^ and Atg21 was more evident by BiFC assays compared to the two-hybrid assays. This is likely because the bimolecular fluorescent complex of YFP^C^-Rho5 and Atg21-VN might be more stable or irreversible compared to the interaction between DBD-Rho5^WT^ and AD-Atg21 in a two-hybrid assay.

Consistent with our initial screen by spot assays, an *atg21*Δ mutant exhibited higher cell survival upon exposure to H_2_O_2_ (Figure 5C). Because the WT *ATG21* strain undergoes little cell death in the absence of Rho5 and vice versa, and because *atg21*Δ cells exhibit little cell death even when *rho5^G12V^* is expressed (see Figure 3Ba), it is likely that Rho5 and Atg21 function together to promote cell death upon exposure to oxidants. Moreover, the interaction between Rho5-GTP and Atg21 described above suggest that Atg21 may function downstream of Rho5. Atg21 is known to be involved in the CVT pathway and autophagy of mitochondrion (Kim and Klionsky 2000). Interestingly, a previous report suggests that cells deleted for *RHO5* or its exchange factor (*dck1* or *lmo1*) exhibit reduced mitophagy (Schmitz *et al.* 2015), supporting the idea that Rho5 may be activated upon exposure to oxidants and promote cell death via Atg21. *ATG18* and *ATG21* are paralogous genes that encode WD-repeat proteins, similar to human WIPI2 (WD repeat domain, phosphoinositide interacting 2) (Dove *et al.* 2004; Strømhaug *et al.* 2004). However, this role of Atg21 in oxidant-induced cell death is unlikely to be shared with Atg18, since *atg18*Δ expressing the GTP-locked Rho5 did not exhibit resistance to H_2_O_2_ (Figure 5D).

The interconnection between apoptosis and autophagy is still poorly understood. Autophagy is generally known to be a cell survival mechanism, but recent studies suggest that autophagy-dependent cell death (ADCD) is a regulated cell death mechanism that requires components of the autophagy machinery instead of other cell death pathways. Although it is more likely that ADCD depends on components of the autophagic machinery rather than autophagic responses, increasing evidence suggests that these pathways are intricately connected (Cooper 2018; Bialik *et al.* 2018; Carmona-Gutierrez *et al.* 2018). Previous studies have shown that the expression of human apoptosis initiator caspase-8 or caspase-10 is toxic to budding yeast by inducing a lethal phenotype with hallmarks of apoptosis and autophagy (Lisa-Santamaría *et al.* 2009). Interestingly, this caspase-10-induced cell death in yeast requires genes involved in autophagy (Lisa-Santamaría *et al.* 2012). While further investigation is required to fully understand how Rho5-GTP promotes cell death via Atg21, our findings suggest an interesting possibility that a Rho GTPase may utilize components of the autophagy machinery to promote cell death under oxidative stress.

In summary, our genome-wide studies reported in this study suggest that Rho5-mediated cell death signaling might impact cellular functions involving vesicle-mediated transport and the plasma membrane. These findings further substantiate the possibility that the oxidant-induced cell death process is complex and requires an interplay of a wide range of intracellular processes. It is tempting to speculate that common cellular components or pathway such as autophagy or eisosomes might be utilized either for cell survival or for death depending on upstream signaling molecules and/or the level of stress. Indeed, despite the similar structure of Rho GTPases, Rho1, which is involved in the cell integrity pathway (Levin 2011), is necessary for survival under oxidative stress (Lee *et al.* 2011), whereas Rho5 is necessary for cell death (Singh *et al.* 2008; this study). To fully understand the functional significance of these interactions and the complex signal integration for survival and death will require further investigation.
